# Opioid Use Disorder and intracerebral hemorrhage in Isfahan, Iran: a case–control study

**DOI:** 10.3389/fneur.2024.1420675

**Published:** 2024-09-16

**Authors:** Mohammad Saadatnia, Razieh Norouzi, Mohammad Amin Najafi, Sahand Gol Parvar, Arash Najafian, Aryan Tabatabei, Melika Foroughi, Sadaf Esteki, Fariborz Khorvash

**Affiliations:** ^1^Department of Neurology, Isfahan Neurosciences Research Center, Alzahra Hospital, Isfahan University of Medical Sciences, Isfahan, Iran; ^2^Department of Medical Science, Islamic Azad University, Najafabad Branch, Isfahan, Iran

**Keywords:** opioid, life style, intracerebral hemorrhage (ICH), opium abuse, case–control analysis

## Abstract

**Background:**

Opium use disorder is a significant health problem in our country, leading to a considerable number of health issues. Opium has several detrimental effects on its consumers. However, the effect of Opium use disorder on intracerebral hemorrhage (ICH) has not been evaluated. This study aims to evaluate the relationship between Opium use disorder and ICH.

**Methods:**

In this case–control study, 402 patients with ICH and 404 patients without ICH enrolled. Opium use disorder, other vascular risk factors including diabetes mellitus, hypertension, hyperlipidemia, and tobacco smoking was compared between these groups. Patients with ICH were divided into two groups; first group are patients with history of Opioid Use Disorder and second group are those patients without Opioid Use Disorder. ICH features including clinical and imaging characteristics and prognostic findings were compared between patients with and without Opium use disorder.

**Results:**

This case–control study of 806 participants found that hypertension (OR = 6.84, 95% CI: 5.03–9.34, *p*-value: <0.001), Opium use disorder (OR = 4.23, 95% CI: 2.42–7.35, *p*-value: <0.001) and tobacco smoking (OR = 1.47, 95% CI: 1.01–2.16, *p*-value: 0.049) had a higher risk of ICH. Opium-addicted subjects had higher ICH scores (2.61 ± 1.27 vs. 2.11 ± 1.29, *p*-value: 0.005), were more likely to have infratentorial hemorrhage (22% vs. 12%, OR = 2.13, 95% CI: 1.06–4.28, *p*-value: 0.038), more likely to be intubated (66% vs. 54%, OR = 1.79, 95% CI: 0.98–3.27, *p*-value = 0.041) and had lower GCS scores (9.58 ± 3.60 vs. 8.25 ± 3.88, *p*-value: 0.01). The effect of Opium use disorder independently on ICH was also shown in logistic regression (adjusted OR = 3.15, *p*-value = 0.001).

**Conclusion:**

This study is the first to evaluate the effect of Opium use disorder on ICH, identifying Opium use disorder as a new potential risk factor for ICH.

## Introduction

Intracerebral hemorrhage (ICH) is a life-threatening emergency requiring immediate medical treatment. The primary risk factor for ICH is high blood pressure (1). Other risk factors include diabetes mellitus, ischemic heart disease, hyperlipidemia, smoking, coagulation disorders, the use of anticoagulant drugs, and blood dyscrasias (2–4). Controlling these risk factors to prevent the onset of ICH and reduce its mortality and morbidity is crucial (5).

The role of substance abuse in cerebrovascular disease has been identified in previous studies. In a study by Saadatnia and colleagues, Opium use disorder was proven to be a risk factor for ischemic stroke (6). Another study by Kurt and colleagues in 1996 identified cocaine use as a major risk factor for ICH (2). Subsequent studies also confirmed the role of amphetamines in the onset of ICH (7).

Opium is the most commonly used drug traditionally in the Middle East, especially in Iran. Derived from the opium poppy plant, it is available in various forms, including raw opium, syrup, heroin, methadone, and morphine. The effect of opium on ischemic stroke has been widely evaluated in previous studies, and a systematic review conducted in 2022 found that Opium use disorder increases both the incidence and mortality of ischemic stroke (8).

The effect of opium use on intracerebral hemorrhage has not yet been evaluated. This case–control study aims to investigate the role of Opium use disorder in the clinical, imaging characteristics, and prognosis of ICH. Additionally, an epidemiological report of patients with ICH hospitalized in two main neurology educational hospitals in Isfahan between 2021 and 2023 is provided in this study.

## Methods

This case–control study was conducted in Isfahan city during the years 2021–2023 by reviewing the existing medical records and files in the medical documents and records department of Al-Zahra and Kashani hospitals for patients with primary and secondary intracranial hemorrhage. A total of 806 participants (402 patients with ICH and 404 controls) were enrolled. Patients in the control group were randomly sampled from patients hospitalized in other departments of the mentioned hospitals (e.g., skin, plastic surgery) who were demographically (age and sex) matched to the patient group.

The data obtained from patients included age, sex, tobacco smoking, blood pressure, drug history, history of hypertension, history of diabetes mellitus, history of hyperlipidemia, ICH subtype (primary or secondary), ICH volume, and its location. Hypertensive patients are considered as patients with either history of hypertension or high blood pressure in admit. Smoking is defined as tobacco use. Subtypes of opium including Taryak, Heroin, Sookhte, Shire and methadone are assessed among patients. Patients with ICH and those without this disease were compared in terms of various risk factors and opium reviews. Patients with ICH with a history of Opioid Use Disorder were compared with patients with ICH who do not use these substances in terms of clinical and laboratory features and prognosis. Opioid Use Disorder is defined as the chronic use of opioids that causes clinically significant distress or impairment. Additionally, all patients with ICH were followed up by phone call 3 months after discharge from the hospital through interview with the patient if he/she able to talk and oriented. If the patient was aphasic, had severe dysarthria or was not oriented to response, the interview was performed with family member/caregiver. The disability status was evaluated through the Modified Rankin Score (MRS) (9, 10). The MRS is a 6-point disability scale with possible scores ranging from 0 to 5, with a separate category of 6 added for patients who expire.

### Statistical analysis

The collected information was entered into SPSS v 22.0 software, and descriptive statistical indices such as mean, standard deviation, frequency, and frequency percentage were used to describe the data. Quantitative results are presented as mean ± standard deviation (SD). For data analysis, independent t-tests, chi-square tests, and if necessary, Fisher’s exact test were used. Logistic regression was performed to evaluate the effects of Opioid Use Disorder on the likelihood of patients having ICH.

## Results

In this study, 402 patients with ICH (239 male and 163 female) and 404 patients without ICH (225 male and 179 female) were surveyed.

Table 1 represents the demographic and clinical characteristics of both groups. The mean age of the ICH group was 62.01 ± 14.85 years, and the control group was 57.86 ± 15.80 years (*p*-value = 0.061). Tobacco smokers had a 1.47 times higher risk of ICH compared to the control group (OR = 1.47, 95% CI: 1.01–2.16, *p*-value: 0.049). Patients with hypertension and Opioid Use Disorder had a significantly higher risk of ICH compared to the control group (OR = 6.84, 95% CI: 5.03–9.34, *p*-value: <0.001; and OR = 4.23, 95% CI: 2.42–7.35, *p*-value: <0.001, respectively). Opium usage types prevalence was: Taryak (*N* = 39.6% vs. 35.2%), methadone (31.7% vs. 29.4%), Shire (9.5% vs. 11.7%), Sookhte (4.7% vs. 5.8%), Heroin (3.1% vs. 5.8%) and using mix types of opioids (11.1% vs. 11.7%). None of these types showed significant differences between two groups.

**Table 1:** Comparing demographic and clinical characteristics of ICH group and control group.

Variable	Group		*p* value	OR (95%CI)
	Case (*n* = 402)	Control (*n* = 404)		
Age (years, mean ± SD)	62.01 ± 14.85	57.86 ± 15.80	0.061	n/a
Sex [male, *n* (%)]	239 (59.45%)	225 (55.69%)	0.280	1.17 (0.88–1.54)
Tobbaco smoking [*n* (%)]	73 (18.16%)	53 (13.12%)	0.049^*^	1.47 (1.01−2.16)
Tobbaco smoking (pack/year, mean ± SD)	2.22 ± 5.31	1.95 ± 7.57	0.562	n/a
Opioid Use Disorder [*n* (%)]	63 (15.67%)	17 (4.21%)	<0.001^*^	4.23 (2.42–7.35)
Hypertension [n (%)]	249 (61.94%)	115 (24.46%)	<0.001^*^	6.84 (5.03–9.34)
Diabetes mellitus [*n* (%)]	81 (20.15%)	69 (17.08%)	0.263	1.22 (0.86–1.75)
Hyperlipidemia [*n* (%)]	77 (19.15%)	66 (16.33%)	0.288	1.13 (0.84–1.74)

^*^Values less than 0.05 considered statically significant; OR, odds ratio; CI, confidence interval; n/a, not applicable.

Table 2 represents a comparison of ICH categories and characteristics between opium-addicted and non-addicted groups. The mean GCS was significantly higher in the patients without opium use disorder (9.58 ± 3.60 vs. 8.25 ± 3.88, *p*-value: 0.01). Patients with opium use disorder had more ICH scores compared to other group (2.61 ± 1.27 vs. 2.11 ± 1.29, *p*-value: 0.005). Furthermore, patients with opium use disorder were more likely to have infratentorial hemorrhage (22% vs. 12%, OR = 2.13, 95% CI: 1.06–4.28, *p*-value: 0.038). Also, frequency of intubation of patients with Opioid Use Disorder was more than patients without Opioid Use Disorder (66% vs. 54%, OR = 1.79, 95% CI: 0.98–3.27, *p*-value = 0.041). Number of deaths and MRS after 3 months were obtained by phone call, 82 patients did not answer and the reported percentages are within 324 patients. Within these 320 patients’ the differences between two groups in terms of total number of death and MRS after 3 (mean ± SD) were not significant (*p* > 0.05). Total number of deaths after 3 months were 57% in patients with opium use disorder and 44% in the other group, (*p* = 0.12). mean ± SD MRS after 3 months were 4.2 ± 2.2 in patients with opium use disorder and 3.8 ± 2.2 in patient without opium use disorder, (*p* = 0.33).

**Table 2:** Comparing ICH categories and characteristics in Opioid Use Disorder and non- Opioid Use Disorder groups.

ICH categories/characteristics	Non - opioid use disorder	Opioid use disorder (*N* = 63)	*p*-value	
	(*N* = 339)			OR (95% CI)
ICH subtype (*n* (%))			0.38	1.6
Primary	303 (89.3%)	54 (85.7%)		
Secondary	36 (10.6%)	9 (14.2%)		
Types of secondary ICH (number)			0.14	n/a
Amyloid angiopathy	4	1		
Cerebral vein thrombosis	1	0		
Hemorrhagic transformation	2	0		
Vascular malformation	8	3		
Coagulopathy	5	2		
Anticoagulation	14	1		
Fibrinolytic therapy	3	3		
Admission GCS (mean ± SD)	9.58 ± 3.60	8.25 ± 3.88	0.01^*^	n/a
ICH volume [milliliters, (mean ± SD)]	34.05 ± 29.58	40.30 ± 28.08	0.11	n/a
ICH score (mean ± SD)	2.11 ± 1.29	2.61 ± 1.27	0.005^*^	n/a
Discharge MRS (mean ± SD)	3.97 ± 1.81	4.25 ± 1.78	0.257	n/a
Follow up MRS (mean ± SD)	4.48 ± 2.37	4.93 ± 2.27	0.15	n/a
ICU hospitalization [days, (mean ± SD)]	8.74 ± 14.2	9.84 ± 13.2	0.55	n/a
IVH [yes, *n* (%)]	187 (55.1%)	42 (66.6%)	0.09	1.56
Hemisphere [*n* (%)]			0.92	0.8
Right	178 (52.5%)	37 (58.7%)		
Left	157 (46.3%)	26 (41.2%)		
ICH location [*n* (%)]			0.038^*^	2.1
Supratentorial	297 (87. 6%)	49 (77.7%)		
Infra tentorial	42 (12.3%)	14 (22.2%)		
Intubation during hospitalization	183 (53.9%)	42 (66.6%)	0.041^*^	1.79
Death in the hospital [*n* (%)]	109 (32.1%)	25 (39.6%)	0.24	1.4
ICH Functional score	7.69 ± 2.29	7.14 ± 2.26	0.056	–

^*^Values less than 0.05 considered statically significant; OR, odds ratio; CI, confidence interval; n/a, not applicable; ICH, intracerebral hemorrhage; GCS, Glasco coma scale; MRS, modified ranking scale; IVH, intraventricular hemorrhage.

The logistic regression model used to evaluate the independent effect of Opioid Use Disorder on ICH showed that patients with Opioid Use Disorder were 3.53 times more likely to have a ICH after adjusting for relevant predictors (*p*-value = 0.001).

## Discussion

Intracerebral hemorrhage (ICH) is an acute condition characterized by the rupture of a blood vessel within the brain, leading to internal bleeding and requiring emergent medical treatment. ICH is the second most common subtype of stroke, and its incidence increases with age and is more common in men. This study is the first to evaluate the effect of Opioid Use Disorder on ICH characteristics.

We focused on the incidence, clinical and laboratory characteristics, mortality, and outcomes of primary and secondary intracranial hemorrhage in patients consuming opium. Iran is grappling with the world’s second most severe opioid addiction problem. According to the World Health Organization (WHO), Iran has the highest rate of opium abusers in the world, with opium use in Iran being three times the global average (11). Efforts should be made to educate the public about the dangers of opium. According to various studies, the prevalence of Opioid Use Disorder in Iran is between 2 to 5% (12–15). However, among the 402 patients enrolled in this study with ICH, 15.6% were opium-addicted. These noticeable differences (15% vs. 2–5%) suggest the possible effect of Opioid Use Disorder on ICH.

The main mechanism of ICH in patients with chronic hypertension is lipohyalinosis and arteriolar dissections (16). The effect of opium on vascular disorders in previous studies is explained by rapid endothelial damage (17). Several mechanisms could be presumed for this relationship. Firstly, opium can affect the coagulation system as it inhibits the production and function of platelets (18) and interferes with clotting factors (19). Secondly, opium can damage the vascular integrity of the brain by causing inflammation, oxidative stress, and apoptosis in the endothelial cells of blood vessels (20). Thirdly, opium reduces the production of nitric oxide, a molecule that protects blood vessels from injury. Fourthly, long-term opium dependency has negative impacts, thus aggravating diabetes, dyslipidemia, and hypertension, which are the most important risk factors for ICH (21). These effects can increase the risk of both ischemic and hemorrhagic stroke. However, the exact mechanisms and causal relationships between Opioid Use Disorder and ICH are not fully understood and require further research.

Our results indicate that subjects with opium use disorder presenting with ICH had lower GCS, more prevalent infratentorial ICH, more requirement for intubation and higher ICH scores. These findings reflect the more severe presentation of ICH in patient with opium use disorder. ICH score is a simple clinical grading scale that allows risk stratification on presentation with ICH. Thirty-day mortality increases as ICH Score increases. Lower GCS and higher rate of intubation in patient with opium use disorder might be due to withdrawal symptoms in these patients. Blood–brain barrier (BBB) in the posterior circulation is generally considered to be more vulnerable compared to the anterior circulation due to several factors; first, *Anatomical and Functional Differences*: The posterior circulation, supplied by the vertebral and basilar arteries, serves critical areas like the brainstem and cerebellum. These regions have a higher metabolic demand and are more susceptible to fluctuations in blood flow and pressure. Second, *autonomic nervous system influence*: the posterior circulation is more influenced by autonomic nervous system which can affect cerebral blood flow and BBB permeability. These factors contribute to a relatively weaker BBB in the posterior circulation, making it more prone to disruptions under pathological conditions such as infratentorial ICH.

## Conclusion

As the first study evaluating the effect of Opioid Use Disorder on intracerebral hemorrhage characteristics, we found that Opioid Use Disorder is more prevalent in patients with ICH compared to the general population. Also, patients with ICH and Opioid Use Disorder had lower GCS, more prevalent infratentorial ICH, more frequent intubation and higher ICH scores compared to ICH patients without Opioid Use Disorder. Thus, according to our findings, we suggest Opioid Use Disorder as a potential risk factor for ICH.

## Limitations

Several limitations exist in this study. *First*, although psychological safety precautions and confidentiality reassurances were provided by the medical team, it is still possible that the stigma of addiction may have affected some patients’ comfort in disclosing all the details of their opium consumption. The *second* limitation is related to the purity of the opium that patients consume, as sometimes there may be impurities combined with opium derivatives. Therefore, it is not possible to fully evaluate the effect of those potential additional ingredients. The *third* limitation is that comprehensive drug toxicology testing was not performed, but the included patient groups disclosed opioid use only. *Forth*, our patients were followed after 3 months to evaluate the number of death and MRS after 3 months. 82 patients did not answer the phone call and thus the statistics about 3-month prognosis of our patients is reported based on 322 patients who answered the phone call. *Fifth*, the validity of Persian version of MRS is not yet assessed and we used the Accurate, semantic and conceptual translation of the English version. Finally, the control group was selected from other patients inside the hospital, who might have more cooperation than controls from outside the hospital.

## Data Availability

The raw data supporting the conclusions of this article will be made available by the authors, without undue reservation.
